# Strain-specific genetic relationships between Mycoplasma synoviae immunity and egg production traits in two layer lines

**DOI:** 10.1016/j.psj.2026.107228

**Published:** 2026-06-03

**Authors:** Xingyue Niu, Qi Yang, Xiaoyu Zhao, Lei Shi, Erying Hao, Yifan Chen, Lanhui Li, Huage Liu, Lijun Xu, Rongyan Zhou, Hui Chen, Xiaorong Zhang, Dehe Wang

**Affiliations:** a College of Animal Science and Technology, Hebei Agricultural University, Baoding Hebei 071001, China; b Baoding Xingrui Agriculture and Animal Husbandry Development Co., Ltd. Baoding 071001, China; c Hebei Province Animal Husbandry and Veterinary Medicine Research Institute. Baoding 071001, China; d Baoding Animal Husbandry Workstation. Baoding 071001, China; e College of Veterinary Medicine, Yangzhou University, Yangzhou 225009, China

**Keywords:** *Mycoplasma synoviae*, Antibody titer, Genetic parameters, Production traits, Egg quality

## Abstract

*Mycoplasma synoviae* (MS) infection causes inflammatory responses in laying hens, leading to significant egg quality deterioration and potential mortality. Due to suboptimal vaccine efficacy and control measures, genetic improvement for disease resistance represents a critical strategy. Using 2,375 Rhode Island Red (RIR) and 2,575 White Leghorn (WL) purebred hens with distinct genetic backgrounds, we systematically evaluated immune responses, production performance, and egg quality throughout the laying cycle following MS immunization. At 100 wk, plasma IgA, IgG, and IgM concentrations were quantified in 720 RIR and 435 WL hens, with egg quality measured in WL hens and multi-trait genetic parameters systematically estimated. Phenotypic analysis revealed that IgA and IgM concentrations were similar between lines (RIR: 1786.8, 748.4 μg/mL; WL: 1793.4, 742.1 μg/mL), while IgG and MS-specific antibody titer (MSAT) were significantly higher in WL than RIR (*P<0.001*), with high variation in immune traits (CV: 27.6%-46.1%). Heritabilities of MSAT were low in both lines (RIR: 0.19; WL: 0.15), and heritabilities of immunoglobulins ranged from 0.09 to 0.17. Genetic correlations revealed positive associations among immunoglobulins in both lines (RIR: 0.42-0.75; WL: 0.38-0.83). Body weight at 18 wk (BW18) was negatively genetically correlated with age at first egg (AFE) in both lines (RIR: -0.57; WL: -0.61). The two lines showed different genetic correlation patterns: MSAT with IgA (RIR: -0.46; WL: 0.16), MSAT with egg production from 301 to 560 d (EP301-560) (RIR: 0.38; WL: -0.24), and MSAT with shank length at 6 wk (SL6) (RIR: -0.29; WL: 0.19). Immunoglobulins were mostly positively correlated with growth traits in RIR but negatively correlated in WL. MSAT was positively genetically correlated with eggshell strength (ESS), eggshell thickness (EST), egg shape index (EI), and eggshell weight (ESW) in WL, indicating a genetic synergy between anti-MS antibody response and eggshell quality. This first estimation of MSAT genetic parameters reveals complex associations among immune, production, growth, and egg quality traits, providing a direct reference for line-specific, multi-trait, synergistically optimized disease-resistant breeding programs.

## Introduction

*Mycoplasma synoviae* (**MS**) is recognized by the World Organization for Animal Health as one of the most pathogenic mycoplasmas affecting poultry production worldwide (WOAH, 2021). This pathogen is transmitted both horizontally and vertically (Feberwee et al., 2017; Ter Veen et al., 2020), causing synovitis (Landman et al., 2012), respiratory disease (Lockaby et al., 1998), and eggshell apex abnormality (**EAA**) syndrome (Feberwee et al., 2009; Jeon et al., 2014). These pathological conditions lead to growth suppression (Landman and Feberwee, 2001), reduced egg production (Kursa et al., 2019), and increased mortality (Ma et al., 2024), resulting in sustained economic losses for the poultry industry (Landman, 2014; Nascimento et al., 2005). MS is highly prevalent worldwide. A multinational meta‑analysis reported a global pooled prevalence of 38.4% for MS infection (Chaidez-Ibarra et al., 2022), while a survey of 463 commercial chicken flocks in China showed a seropositivity rate as high as 80.99% (Xue et al., 2017), underscoring the severe challenges for disease prevention and control. Molecular epidemiological studies further demonstrate that MS exhibits host specificity across different avian species and is frequently involved in co-infections with other pathogens, such as Newcastle disease virus (**NDV**) and infectious bronchitis virus (**IBV**) (Hutton S et al., 2017; Landman WJ and Feberwee A, 2004), which exacerbates disease severity and complicates control measures. Consequently, MS infection has emerged as a critical health and economic constraint on the sustainable development of the global layer industry (Feberwee et al., 2025).

Current MS control strategies in the poultry industry rely primarily on biosecurity (Yadav et al., 2022), antibiotics (Kreizinger et al., 2017), and vaccination (Wu et al., 2024). However, all these strategies face substantial limitations. Long-term use of antibiotics carries risks of antimicrobial resistance and drug residues (Bekő et al., 2020; Zhang et al., 2022). Although macrolides and tetracyclines can alleviate clinical symptoms, they cannot completely eradicate infection (Lysnyansky et al., 2015; Puvača et al., 2020). Studies have shown an increasing trend in the minimum inhibitory concentrations (**MICs**) of tilmicosin and tylosin against MS isolates (Catania et al., 2019; Morrow et al., 2020). Meanwhile, conventional inactivated vaccines still face challenges in overcoming antigenic variation in MS and providing stable and broad cross‑protection (Sun et al., 2024; Yi et al., 2025).

These challenges are rooted in the high genetic diversity of MS itself. The MS genome is approximately 819 kb, encoding about 670 protein-coding genes (Xu et al., 2020a). Among them, the *vlhA* gene, with its 5′ hypervariable region rich in sequence diversity, is an ideal typing target. Based on sequence analysis of its 5′ hypervariable region (76-421 bp), at least 17 genotypes (A-K) have been identified, with genotype K predominating in Chinese field isolates (79.5%) (Bencina, 2002; Sun et al., 2017). The 16S rRNA can also be used for MS strain typing. Buim et al. (2010) sequenced partial 16S rRNA of 19 MS strains and found up to six polymorphic sites among them. Ramirez et al. (2011) used the 16S‑23S rRNA intergenic spacer region and resolved isolates into 10 groups with a minimum inter‑group similarity of 95.8%, indicating limited resolution. Moreover, MS exhibits strong mutability. Three Indian field isolates showed point mutation rates of 12%, 5.1% and 1.8%, respectively, in the *vlhA* gene compared with the reference strain KC506806 (Sumitha et al., 2018). After replicating in chickens for 8 weeks and 5 months, the *vlhA* sequences of MS isolates retained only 65-80% identity with the original inoculum (Slavec, B., et al., 2011). These data suggest the high mutability of the MS genome and its mutational potential. Therefore, relying solely on external interventions may not be sufficient to eradicate MS; genetic improvement of host innate resistance represents a promising direction for sustainable poultry production.

The magnitude and efficacy of the host immune response to MS infection are central determinants of disease outcome. Studies have shown that the pathogen initiates infection via various immunogenic adhesion proteins, including elongation factor Ts (**EF-Ts**; Zhao et al., 2024), elongation factor G (**EF‑G**; Xu et al., 2024), NADH oxidase (**NOX**; Hu et al., 2022), and dihydrolipoamide dehydrogenase (**DLD**; Qi et al., 2022). The host activates specific humoral immunity by recognizing surface antigens represented by the variable lipoprotein haemagglutinin (**VlhA**) system, leading to the production of neutralizing antibodies (Khiari et al., 2012). MS infection also induces high expression of pro-inflammatory cytokines such as IL-1β and IL-6, as well as pattern recognition receptors including TLR1, TLR2, and TLR15 (Xu et al., 2020b). In turn, MS achieves immune evasion through high‑frequency antigenic variation of the VlhA system (Bencina, 2002). Despite extensive studies on pathogen biology and vaccine development (Feberwee et al., 2022), comparative studies on the differences in anti‑MS immune responses among different lines from the perspective of host genetics remain scarce.

Indeed, it is well established that host genetic variation influences antibody responses in various livestock species. In chickens, heritabilities for innate and adaptive immune traits range from 0.06 to 0.53 (Emam et al., 2014). Divergent selection experiments have directly demonstrated the feasibility of genetic improvement: after nine generations of selection for antibody titers against sheep red blood cells (**SRBC**), realized heritabilities were 0.21 and 0.25 for the high and low lines, respectively (Pinard et al., 1992); after 18 generations, the genetic difference between the two lines reached 5.1 phenotypic standard deviations (Bovenhuis et al., 2002). For natural antibodies (**NAb**) binding keyhole limpet hemocyanin (**KLH**), heritabilities are 0.14 for IgM, 0.10 for IgA, and 0.07 for IgG, with a maternal environmental effect of 0.06 for IgM (Berghof et al., 2015). For pathogen-specific responses, heritabilities of antibody titers against Newcastle disease virus (**NDV**) range from 0.22 to 0.28 (Hako Touko et al., 2021), and genomic selection has been successfully applied to improve NDV and avian influenza virus antibody responses (Liu et al., 2014). These findings indicate that antibody-related traits are under moderate genetic control in poultry. However, key genetic parameters for resistance to MS have not been reported, including the heritability of MS-specific antibodies and other immune traits, their genetic correlations with economically important traits, and the heterogeneity of the genetic architecture of MS resistance among chicken lines with different genetic backgrounds. This knowledge gap limits the effective integration of MS resistance into modern breeding programs.

Accordingly, this study shifts the research focus from pathogen‑targeted intervention to host‑centered investigation. We performed a systematic comparative genetic analysis using two commercial layer lines with distinct genetic backgrounds and selection histories: Rhode Island Red (**RIR**) and White Leghorn (**WL**). The core objectives of this study were: 1) To systematically estimate and compare the heritabilities of MS‑specific antibodies titer (**MSAT**) and conventional immunoglobulin isotypes (IgA, IgG, IgM) in both lines for the first time; 2) To construct genetic and phenotypic correlation matrices between these immune traits and key economic traits, including growth performance, laying persistency, and egg quality. The results of this study are expected to establish an important genetic parameter foundation for breeding poultry resistant to MS, and provide a scientific basis for line‑specific precision breeding.

## Materials and methods

### Ethics statement

The use of research animals and the corresponding care practices were conducted in compliance with national and institutional guidelines, and approved by the Animal Care and Use Committee of Hebei Agricultural University (Approval Number: 2024021).

### Experimental animals

The study population comprised 2,375 pureline Rhode Island Red (**RIR**) and 2,575 pureline White Leghorn (**WL**) hens. The hens were supplied by Baoding Xingrui Agricultural and Animal Husbandry Development Co., Ltd. (Baoding, China), and the experimental period spanned from 6 to 100 wk of age. Hens from both genetic lines were housed together in the same fully environmentally controlled poultry house. A four-tier H-type caging system (Model: 9LCDd-348; YANBEI Group., Beijing, China) was employed. Stocking density was maintained at 20 to 25 hens per square meter from 6 to 12 wk of age. From 13 wk through to 100 wk of age, each hen was housed individually in a single cage. To guarantee that both lines experienced identical environmental conditions, including light, airflow, temperature, and humidity, hens were randomly distributed across cage tiers and positions within the house. Throughout the trial, all hens were fed age-appropriate diets and had ad libitum access to feed and water. The photoperiod was regulated according to the following schedule: from 6 to 17 wk of age, an 11 h light period (07:00 to 18:00) at an intensity of 5 lux was provided. During 18 to 22 wk, the lights‑off time was progressively delayed by 0.5 h each week, starting from an initial time of 18:00. From 23 to 27 wk of age, the lights‑on time was gradually advanced by 0.5 h each week, starting from 07:00, thereby extending the total daily photoperiod to 16 h. From 28 to 100 wk, an intermittent lighting schedule of 1 h light (00:00 to 01:00) : 4 h dark : 15 h light : 4 h dark (1L:4D:15L:4D) was implemented, with a light intensity of 10 lux. The entire flock was vaccinated against *Mycoplasma synoviae*. At 18 d of age, each hen received an ocular drop administration of 0.03 ml (containing ≥ 1.0 × 10^8^ CCU/ml) of the MS-H strain live attenuated vaccine (Import Veterinary Drug Registration Certificate No. 2022 Wai Shou Yao Zheng Zi 50; Bioproperties Pty. Ltd., Ringwood, VIC, Australia). At 90 d of age, each hen received a subcutaneous injection in the neck region of 0.5 ml (containing ≥ 1.9 × 10^9^ CCU/ml) of the YBF-MS1 strain inactivated vaccine (Veterinary Drug License No. 150132337; Qingdao Yibang Bioengineering Co., Ltd., Qingdao, China). The complete vaccination schedule of the experimental flock is provided in Supplementary Table S1. Environmental control for both the brooding and laying periods was managed using a Little Giant intelligent climate control system (Model LG800; Little Giant Husbandry Equipment Co., Ltd., Chengdu, China). From 6 to 8 wk of age, the house temperature was maintained within the range of 20-25°C with a relative humidity of 45%−55%. From 9 to 100 wk of age, the temperature was maintained between 18 and 20°C, and relative humidity was maintained at 30%−50%.

### Data collection

***Growth Trait Measurements****.* Individual body weights of hens from both the RIR and WL lines were measured at 6, 18, and 72 wk of age using a fully automatic, internally calibrated electronic platform scale with a precision of 0.1 g (Model: XY09-XY30MTB); these measurements were recorded as BW6, BW18, and BW72, respectively. Shank length was measured for each hen at 6 and 18 wk of age using an electronic digital caliper with a resolution of 0.01 mm (Model: 500-196-30; Mitutoyo Corporation, Kawasaki, Japan), recorded as SL6 and SL18.

***Egg Production Recording.*** Age at first egg (**AFE**) was recorded for each hen using an intelligent mobile terminal (Model: ZKC 3503-M; Shenzhen Zhigulian Software Technology Co., Ltd., Shenzhen, China). Given the nonlinear characteristics of the laying curve (Bell, 1992), total egg production was partitioned into two phases: from AFE to 300 d of age (**EP300**), reflecting the peak rate of lay, and from 301 to 560 d of age (**EP301**-**560**), reflecting laying persistency. Body weight at first egg (**BW-AFE**) and first egg weight (**FEW**) were also recorded for each hen.

***Blood Collection and Immunoglobulin Assays****.* Based on previous studies, plasma immunoglobulin detection was performed in the WL and RIR lines at 100 wk of age. This time point is well beyond the duration of vaccine immunity (Jones et al., 2006; Markham et al., 1998; Yang et al., 2025) and therefore primarily reflects the immune responses of the flocks to natural field exposure, which is more consistent with practical production conditions. Furthermore, the Chinese Ministry of Agriculture and Rural Affairs has proposed that high-yielding laying hens should produce more than 500 eggs by 100 wk of age, which requires extending the laying cycle. Conducting measurements at this stage meets both national and industry development needs and also reflects the relationships among late-stage antibody levels, production performance, and growth traits throughout the entire laying period. At 100 wk of age, wing vein blood samples were collected from RIR (n = 720) and WL (n = 628) hens into 5 mL EDTA anticoagulant tubes (Hebei Kangweishi Medical Technology Co., Ltd., Shijiazhuang, China). Plasma was separated by centrifugation using a benchtop high-speed refrigerated centrifuge (Model: TGL-16.5M; Shanghai Luxiangyi Centrifuge Instrument Co., Ltd., Shanghai, China) and stored for subsequent immunological assays. Concentrations of IgA, IgG, and IgM in plasma were determined using commercial 96T chicken-specific ELISA kits (IgA: Model YX-090701C; IgG: Model YX-090707C; IgM: Model YX-090713C; Beijing Borui Science & Technology Co., Ltd., Beijing, China). Assays were performed on plasma samples from RIR (n = 720) and WL (n = 435) hens at 100 wk of age. For each plate, absorbance was measured at 450 nm using a multifunctional microplate reader (Model: Flash SuPerMax 3100; Shanghai Shanpu Biotechnology Co., Ltd., Shanghai, China). A standard curve was constructed using six standards, and sample concentrations were calculated provided that the coefficient of determination (R²) of the standard curve exceeded 0.9.

***MS‑specific antibody titer (MSAT) measurement****.* MSAT were quantitatively measured in plasma samples from RIR (n = 720) and WL (n = 628) hens at 100 wk of age using a commercial 96T ELISA kit (Product No.: 99-06728; IDEXX Laboratories, Inc., Beijing, China). MSAT was measured using an indirect Enzyme-linked immunosorbent assay (ELISA). Each plate included two negative controls (NC) and two positive controls (PC), as well as test samples. Samples were diluted 500-fold using a two-step dilution procedure prior to addition to antigen-coated plates. Aliquots (100 µL) of diluted samples, undiluted NC, and undiluted PC were added to the wells of the antigen‑coated plate. After incubation at 18-26 °C for 30 min, the wells were washed 3-5 times with approximately 350 µL of wash solution per well and tapped dry. Then, 100 µL of enzyme‑labeled antibody was added, followed by a second incubation at 18-26 °C for 30 min, after which the wells were washed and tapped dry again. Next, 100 µL of 3,3′,5,5′-tetramethylbenzidine (TMB) substrate solution was added and incubated at 18-26 °C for 15 min. The reaction was terminated by adding 100 µL of stop solution to each well. Absorbance was measured at 650 nm (A_650_) using the microplate reader. For result validation, the mean absorbance of the negative controls (NC_x_ = [NC_1_A_650_+ NC_2_A_650_] / 2) and positive controls (PC_x_ = [PC_1_A_650_ + PC_2_A_650_] / 2) was calculated. The assay was considered valid if PC_x_ - NC_x_ > 0.075 and NC_x_ ≤ 0.150. The relative level of MS antibodies in each sample was expressed as the sample-to-positive (S/P) ratio, calculated as: S/P = (Sample A_650_ - NC_x_) / (PC_x_ - NC_x_). The S/P ratio was then converted to an endpoint titer (relative to the 1:500 dilution) using the empirical formula provided by the manufacturer: Log_10_ titer = 1.09 × (Log_10_ S/P) + 3.36. This calculation yielded the MSAT for each individual hen.

***Egg Quality Assessment.*** A total of 1145 eggs were collected on a single day from WL hens at 100 wk of age for egg quality evaluation. The measured traits included egg shape index (**EI**), egg weight (**EW**), eggshell strength (**ESS**), Haugh unit (**HU**), yolk color (**YC**), yolk weight (**YW**), eggshell weight (**ESW**), eggshell thickness (**EST**), and eggshell membrane thickness (**ESMT**). All measurements followed the methodology detailed in a previous publication from our research group (Ren et al., 2023). Considering that previous records showed that egg quality in the RIR line is slightly affected by MS infection and that the WL line as a long-term selected specialized egg-type breed is more representative, egg quality traits were measured only in the WL line. Following phenotypic data collection, genetic parameters for all measured traits were estimated using pedigree information and phenotypic records for each line.

### Statistical analysis

Data were initially organized using Excel 2016. Outliers for each phenotypic trait were identified and removed using the interquartile range (**IQR**) method, with outliers defined as values falling below Q1 - 1.5 × IQR or above Q3 + 1.5 × IQR. All statistical analyses were performed using R software (version 4.3.1) to compare phenotypic differences between the RIR and WL lines.

Prior to group comparisons, normality was assessed using the Shapiro-Wilk test, and homogeneity of variances was evaluated using Levene's test. For data following a normal distribution and meeting the assumption of homogeneity of variances, comparisons between lines were conducted using independent samples *t*‑tests. When variances were unequal, Welch's *t*‑test was applied. For data not conforming to a normal distribution, the Mann-Whitney U test (also referred to as the Wilcoxon rank‑sum test) was employed. To quantify the magnitude of differences between lines, effect sizes were calculated: Cohen's *d* was used for parametric tests, and the rank‑biserial correlation (*δ*) was used for non‑parametric tests. The significance level for all statistical tests was set at α = 0.05.

Based on pedigree information, variance components for all traits were estimated separately for the RIR and WL lines using the restricted maximum likelihood (**REML**) method implemented in the DMUAI module of the DMU software package (DMUv6; University of Aarhus, Denmark). The following animal model was fitted:

y=Xb+Zu+e

where y is the vector of phenotypic observations; **X** and **Z** are the design matrices for fixed effects and the incidence matrix for random additive genetic effects, respectively; **b** is the vector of fixed effects, including the overall mean and batch effects for trait measurements; **u** is the vector of individual additive genetic effects (random); and **e** is the vector of random residual effects. The random effects were assumed to follow a normal distribution: **u** ∼ N ($0,\;A\sigma_{u}^{2}$) and **e** ∼ N ($0,\;I\sigma_{e}^{2}$), where **A** is the additive genetic relationship matrix, **I** is an identity matrix, $\sigma_{u}^{2}$ and $\sigma_{e}^{2}$ are the additive genetic and residual variances, respectively. This normality assumption underlies the REML estimation method and is independent of the distributional properties of the phenotypic observations themselves. Phenotypic correlations between different traits were estimated and tested for significance using Spearman's rank correlation coefficients. As the hens were of the same age and reared under consistent environmental conditions, these factors were not included as fixed or random effects in the model.

## Results

### Comparative phenotypic characteristics between the two lines under MS-specific antibody positive status

Under MS-specific antibody positive status, RIR and WL hens exhibited significant differences in immune, production, and growth traits, with results presented in Table 1. For immune traits, WL hens displayed significantly higher plasma IgG concentrations and MSAT than RIR hens (*P < 0.001*), although the effect sizes were small (*δ* = 0.36 and 0.08, respectively), suggesting limited biological magnitude of the statistical differences. No significant differences were observed between the two lines for IgA and IgM concentrations. All immune traits exhibited high phenotypic coefficients of variation, ranging from 27.6% to 46.1%. AFE was highly significantly earlier in WL hens compared to RIR hens *(P < 0.001*), with a moderate effect size (*δ* = 0.51), reflecting the early-maturing characteristics of WL as a modern high-producing layer line. In contrast, RIR hens displayed highly significantly greater values than WL hens (*P < 0.001*) for FEW, BW-AFE, and all body weight and shank length measurements at the ages assessed, with effect sizes ranging from large to extremely large. Regarding egg production patterns, RIR hens exhibited a small effect size advantage in EP300, whereas WL hens surpassed RIR in EP301-560. This indicates that RIR hens possess superior early laying peak performance, while WL hens demonstrate enhanced late-phase laying persistency.

**Table 1:** Phenotypic comparisons between Rhode Island Red (RIR) and White Leghorn (WL) hens for key traits.

Variable	Line	N	Mean ± SD	Median (IQR)	CV (%)	Test (*P*-value)	Effect Size (Magnitude)
**Immune Traits**							
IgA (㎍/mL)	RIR	700	1786.8 ± 673.2	1692 (1235-2159)	37.7	Wilcoxon (0.326)	*δ* = −0.03 (negligible)
	WL	423	1793.4 ± 752.3	1600 (1284-2018)	41.9		
IgG (㎍/mL)	RIR	708	2316.4 ± 899.7	2111 (1716-2617)	38.8	Wilcoxon (<0.001)	*δ* = 0.36 (small)
	WL	424	2705.0 ± 1167.2	2777 (2395-3098)	43.2		
IgM (㎍/mL)	RIR	708	748.4 ± 319.8	649 (568-791)	42.7	Wilcoxon (0.384)	*δ* = 0.02 (negligible)
	WL	424	742.1 ± 341.9	653 (541-837)	46.1		
MSAT	RIR	721	9216.6 ± 3923.4	8878 (6759-10871)	42.6	Wilcoxon (0.009)	*δ* = 0.08 (negligible)
	WL	623	9275.9 ± 2563.4	9254 (7538-10540)	27.6		
**Production Traits**							
AFE (d)	RIR	2346	134.2 ± 8.2	134 (129-139)	6.1	Wilcoxon (<0.001)	*δ* = 0.51 (medium)
	WL	2528	129.6 ± 10.0	129 (123-135)	7.7		
FEW (g)	RIR	2346	42.5 ± 4.9	42 (39-46)	11.5	Wilcoxon (<0.001)	*δ* = 1.44 (large)
	WL	2520	36.0 ± 4.0	36 (33-39)	11.1		
BW–AFE (g)	RIR	2346	1802.1 ± 128.8	1796 (1713-1885)	7.2	*t*-test (<0.001)	*d* = 1.60 (large)
	WL	2528	1783.1 ± 210.4	1765 (1645-1908)	11.8		
EP300 (eggs)	RIR	2240	159.2 ± 11.0	160 (152-167)	6.9	Wilcoxon (<0.001)	*δ* = 0.30 (small)
	WL	2268	154.0 ± 21.4	158 (141-169)	13.9		
EP301–560 (eggs)	RIR	2092	199.3 ± 47.8	217 (207-228)	24.0	*t*-test (<0.001)	*d* = −0.26 (small)
	WL	1894	208.5 ± 24.9	215 (202-225)	11.9		
**Growth & Body Traits**							
BW6 (g)	RIR	2341	511.3 ± 37.3	510 (486-535)	7.3	*t*-test (<0.001)	*d* = 2.91 (very large)
	WL	2560	413.2 ± 29.8	412 (393-432)	7.2		
BW18 (g)	RIR	2372	1626.3 ± 142.4	1626 (1525-1720)	8.8	*t*-test (<0.001)	*d* = 2.50 (very large)
	WL	2566	1294.4 ± 121.5	1302 (1210-1385)	9.4		
BW72 (g)	RIR	1470	2129.5 ± 230.8	2120 (1956-2292)	10.8	*t*-test (<0.001)	*d* = 1.60 (large)
	WL	1945	1983.1 ± 210.8	1965 (1845-2008)	11.8		
SL6 (mm)	RIR	2341	73.1 ± 2.5	73 (72-75)	3.4	*t*-test (<0.001)	*d* = 1.52 (large)
	WL	2560	69.5 ± 2.1	70 (68-71)	3.1		
SL18 (mm)	RIR	2338	98.5 ± 2.5	99 (97-100)	2.5	*t*-test (<0.001)	*d* = 0.85 (large)
	WL	2566	94.6 ± 6.0	95 (92-98)	6.3		

^1^ Data are presented as mean ± standard deviation (SD) and median (interquartile range, IQR). CV, coefficient of variation.

^2^ Abbreviations and Definitions: IgA, IgG, IgM, concentration of immunoglobulin A, G, and M in plasma (㎍/mL); MSAT, *Mycoplasma synoviae*-specific antibody titer; AFE, age at first egg (d); FEW, egg weight at first egg (g); BW-AFE, body weight at first egg (g); EP300, total number of eggs laid from age at first egg to 300 days of age (eggs); EP301–560, total number of eggs laid from 301 to 560 days of age (eggs); BW6, BW18, BW72, body weight at 6, 18, and 72 wk of age (g); SL6, SL18, shank length at 6 and 18 wk of age (mm).

^3^ Statistical Analysis: Inter-line comparisons were performed using the Mann-Whitney U test (Wilcoxon rank-sum test) or the independent samples *t*-test, as indicated. Exact *P*-values are reported in parentheses.

^4^ Effect Size: Effect size is reported as Cohen‘s *d* for the *t*-test and the rank-biserial correlation (*δ*) for the Wilcoxon test. The magnitude of the effect is interpreted with reference to the conventional benchmarks proposed by Cohen (1988): <0.2 (negligible), 0.2–0.5 (small), 0.5–0.8 (medium), and ≥0.8 (large). For descriptive purposes, Cohen‘s *d* values exceeding 2.0 are additionally noted as “very large”.

### Heritability estimates for immune, production, and growth traits

Variance component analyses based on a large-scale sample enabled estimation of heritabilities for key traits in both lines under MS-specific antibody positive status, with results presented in Table 2. For the core immune indicator of infection, MSAT exhibited low but considerable heritability in both lines (RIR: 0.19 ± 0.08; WL: 0.15 ± 0.10), whereas heritability estimates for conventional immunoglobulins (IgA, IgG, IgM) were lower, ranging from 0.09 to 0.17. Among production traits, AFE displayed moderate to high heritability in both lines (RIR: 0.58 ± 0.05; WL: 0.52 ± 0.05), indicating that this trait is under relatively strong additive genetic control and possesses substantial potential for genetic improvement. FEW and egg production (EP300, EP301-560) exhibited low to moderate heritabilities, ranging from 0.12 to 0.39. Notably, the heritability estimate for EP300 in the WL line (0.12 ± 0.03) was substantially lower than that in the RIR line (0.32 ± 0.04), potentially reflecting partial depletion of additive genetic variance for this trait in WL resulting from long-term intensive selection for egg production. Early growth and development traits displayed high heritabilities in both lines (BW6: 0.74-0.81; SL6: 0.72-0.89), confirming that early growth rate is under strong genetic control. BW72 showed considerable line differences, with low heritability in RIR (0.15 ± 0.05) but moderate to high heritability in WL (0.58 ± 0.06). This suggests that body weight maintenance during the late phase in the dual-purpose RIR line is more prone to environmental effects, whereas adult body weight in WL rests upon a more stable genetic basis.

**Table 2:** Heritability estimates of immune, production, and growth traits in Rhode Island Red (RIR) and White Leghorn (WL) hens.

Line Trait	RIR				WL		
	N	h²	SE		N	h²	SE
IgA	700	0.17	0.09		423	0.16	0.14
IgG	708	0.12	0.08		424	0.13	0.14
IgM	708	0.12	0.09		424	0.09	0.15
MSAT	721	0.19	0.08		623	0.15	0.10
AFE	2346	0.58	0.05		2528	0.52	0.05
FEW	2346	0.39	0.05		2520	0.35	0.05
BW-AFE	2346	0.56	0.05		2528	0.61	0.05
EP300	2240	0.32	0.04		2268	0.12	0.03
EP301–560	2092	0.38	0.05		1894	0.31	0.05
BW6	2341	0.81	0.05		2560	0.74	0.05
BW18	2372	0.55	0.05		2566	0.56	0.05
BW72	1470	0.15	0.05		1945	0.58	0.06
SL6	2341	0.89	0.05		2560	0.72	0.05
SL18	2338	0.65	0.05		2566	0.71	0.05

^1^ N represents the effective sample size for heritability calculation, h² denotes heritability, and SE stands for standard error.

^2^ Abbreviations and Definitions: IgA, IgG, IgM, concentration of immunoglobulin A, G, and M in plasma (㎍/mL); MSAT, *Mycoplasma synoviae*-specific antibody titer; AFE, age at first egg (d); FEW, egg weight at first egg (g); BW-AFE, body weight at first egg (g); EP300, total number of eggs laid from age at first egg to 300 days of age (eggs); EP301–560, total number of eggs laid from 301 to 560 days of age (eggs); BW6, BW18, BW72, body weight at 6, 18, and 72 wk of age (g); SL6, SL18, shank length at 6 and 18 wk of age (mm).

### Genetic and phenotypic correlation networks among traits

**Fig. 1:**
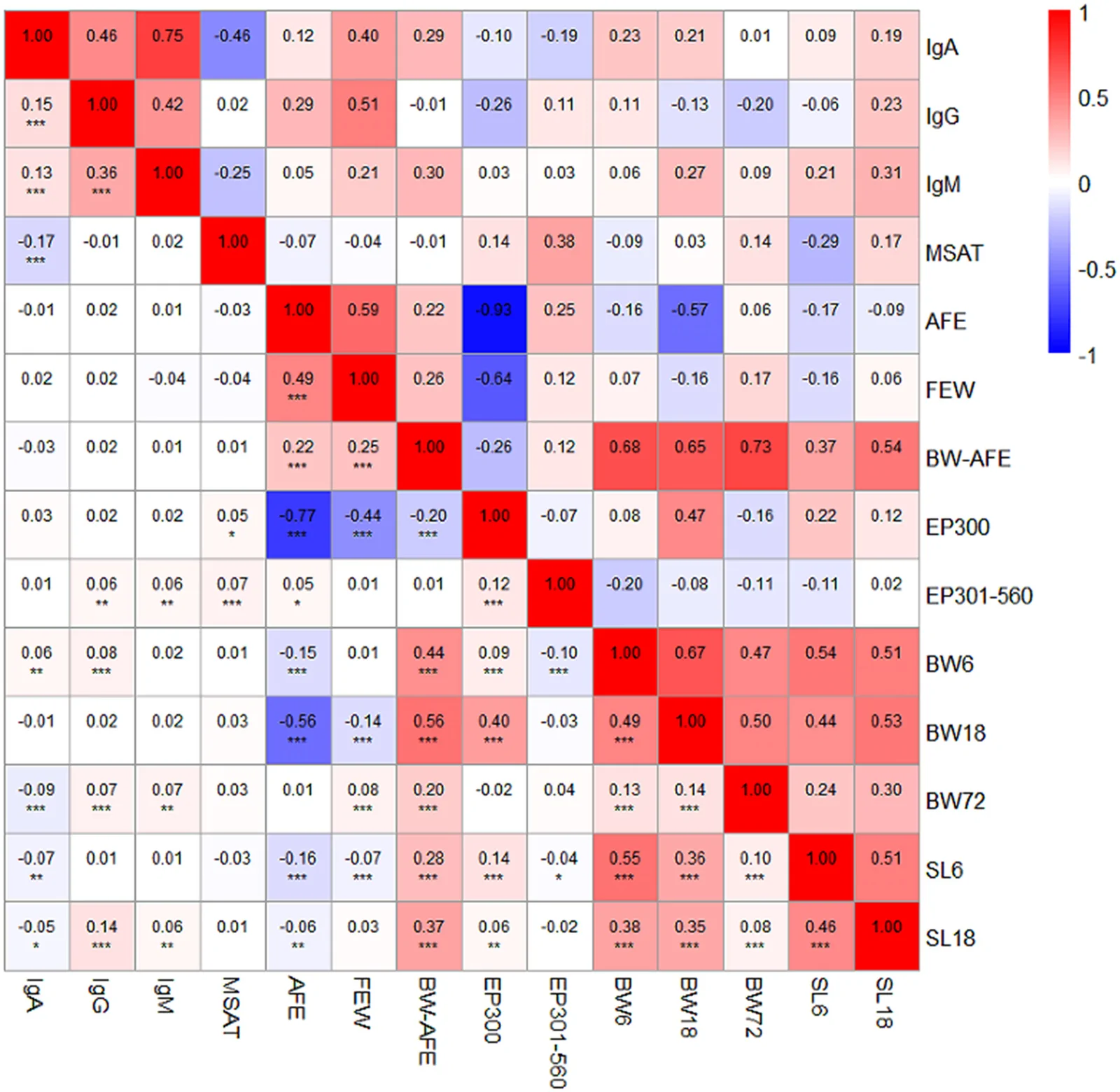
Genetic and phenotypic correlations among immune, production, and growth traits in Rhode Island Red (RIR) hens. ^1^ Abbreviations and Definitions: IgA, IgG, IgM, concentration of immunoglobulin A, G, and M in plasma (㎍/mL); MSAT, *Mycoplasma synoviae*-specific antibody titer; AFE, age at first egg (d); FEW, egg weight at first egg (g); BW-AFE, body weight at first egg (g); EP300, total number of eggs laid from age at first egg to 300 days of age (eggs); EP301–560, total number of eggs laid from 301 to 560 days of age (eggs); BW6, BW18, BW72, body weight at 6, 18, and 72 wk of age (g); SL6, SL18, shank length at 6 and 18 wk of age (mm). ^2^ The upper triangular matrix displays genetic correlation coefficients, reflecting the genetic associations between traits. The lower triangular matrix shows phenotypic correlation coefficients, representing phenotypic associations. ^3^ Statistical significance is indicated by asterisks: * for *P* < 0.05, ** for *P* < 0.01, and *** for *P* < 0.001. ^4^ The color gradient from red to blue represents the transition of correlation coefficients from positive to negative.

***Correlation Characteristics in the RIR Line.*** To elucidate the intrinsic relationships among immune, production, and growth traits under MS-specific antibody positive status, genetic and phenotypic correlation coefficients were estimated for the RIR line, with results presented in Fig. 1. Genetic correlations were generally stronger than their phenotypic counterparts in the RIR line. Positive genetic correlations (r_g_ = 0.42-0.75) and highly significant positive phenotypic correlations (r_p_ = 0.13-0.36, *P < 0.001*) were observed among IgA, IgG, and IgM. In contrast, MSAT exhibited negative genetic correlations with IgA (r_g_ = −0.46) and IgM (r_g_ = −0.25), as well as a significant negative phenotypic correlation with IgA (r_p_ = −0.17, *P < 0.001*), suggesting that the specific immune response to MS operates through distinct genetic regulatory pathways.

Genetic and phenotypic correlations between MSAT and production traits, with the exception of egg production, were generally weak. MSAT displayed positive correlations with EP300 (r_g_ = 0.14; r_p_ = 0.05, *P < 0.05*) and EP301-560 (r_g_ = 0.38; r_p_ = 0.07, *P < 0.001*), but a negative genetic correlation with SL6 (r_g_ = −0.29). These findings imply that, in the RIR line, the genetic potential for an efficient antibody response to MS may be synergistically associated with enhanced laying persistency, albeit at the expense of genetic resources allocated to skeletal growth during the early chick stage. With the exception of a negative genetic correlation between IgG and both BW18 and BW72, genetic correlations between conventional immunoglobulins (IgA, IgG, IgM) and growth traits were predominantly positive. Specifically, both IgG (r_g_ = 0.23; r_p_ = 0.14, *P < 0.001*) and IgM (r_g_ = 0.31; r_p_ = 0.06, *P < 0.01*) were positively correlated with SL18, indicating a potential genetic synergy between robust basal immune status and overall health and developmental capacity. However, inconsistencies between the direction of genetic and phenotypic correlations for some immune-growth trait pairs suggest that environmental effects may dominate phenotypic expression, potentially masking underlying antagonistic genetic relationships between immunity and growth.

Among production traits, AFE was positively correlated with FEW (r_g_ = 0.59; r_p_ = 0.49, *P < 0.001*) and BW-AFE (r_g_ = 0.22; r_p_ = 0.22, *P < 0.001*), but both FEW and BW-AFE were negatively correlated with EP300 (FEW: r_g_ = −0.64; r_p_ = −0.44, *P < 0.001*; BW-AFE: r_g_ = −0.26; r_p_ = −0.20, *P < 0.001*). A later AFE was positively associated with superior late-phase laying persistency (EP301-560: r_g_ = 0.25; r_p_ = 0.05, *P < 0.05*). These associations suggest that, within the RIR line, individuals with a later onset of laying tend to allocate more resources to individual egg weight and body weight, albeit at the cost of reduced early egg production, thereby achieving enhanced laying persistency during the later phase.

Growth and developmental traits were highly correlated among themselves. Positive correlations were observed among BW-AFE, BW6, BW18, BW72, SL6, and SL18 (r_g_ = 0.24-0.73; r_p_ = 0.08-0.56, *P < 0.001*). Regarding correlations between growth and production traits, BW18 was negatively correlated with AFE (r_g_ = −0.57; r_p_ = −0.56, *P < 0.001*), indicating that individuals with greater body weight at 18 wk of age reached sexual maturity earlier. Conversely, BW6 was negatively correlated with EP301-560 (r_g_ = −0.20; r_p_ = −0.10, *P < 0.001*), suggesting that excessive body weight at 6 wk of age may be detrimental to maintaining late-phase laying persistency. This finding highlights the importance of considering specific developmental windows when selecting for early growth traits.

***Correlation Characteristics in the WL Line.*** Similar to the findings in the RIR line, synergies among conventional immunoglobulins were also observed in the WL line, whereas the genetic associations between MSAT and conventional immunoglobulins were generally weak or negative. A very strong positive genetic correlation was detected between IgA and IgM (r_g_ = 0.83), accompanied by a highly significant positive phenotypic correlation (r_p_ = 0.75, *P < 0.001*). MSAT exhibited a positive genetic correlation with IgA (r_g_ = 0.16) but showed highly significant negative phenotypic correlations with both IgA and IgM (r_p_ = −0.09, *P < 0.001*).

In stark contrast to the results observed in the RIR line, MSAT in the WL line displayed strong genetic antagonisms with production traits. MSAT was positively correlated with AFE (r_g_ = 0.47; r_p_ = 0.15, *P < 0.001*), FEW (r_g_ = 0.31; r_p_ = 0.11, *P < 0.001*), and BW-AFE (r_g_ = 0.29; r_p_ = 0.06, *P < 0.001*), while exhibiting negative genetic correlations with EP300 (r_g_ = −0.40) and EP301-560 (r_g_ = −0.24). These findings indicate that, in the WL line, individuals with genetically superior antibody responses tend to reach sexual maturity later and possess lower egg production potential, reflecting a genetic resource competition between disease resistance and high productivity.

Furthermore, the correlation patterns between immune and growth traits in the WL line also diverged from those in the RIR line. MSAT was positively correlated with BW6 (r_g_ = 0.26; r_p_ = 0.15, *P < 0.001*) and SL6 (r_g_ = 0.19; r_p_ = 0.13, *P < 0.001*), indicating a genetic synergy between MSAT levels and early chick growth and development. In contrast, genetic correlations between conventional immunoglobulins (IgA, IgG, IgM) and growth traits were predominantly negative, opposite to the pattern observed in the RIR line. IgM exhibited negative genetic correlations with all growth and developmental traits measured in this study (r_g_ ranging from −0.08 to −0.24). IgA showed negative genetic correlations with all production and growth traits except BW72 (r_g_ ranging from −0.15 to −0.21), and IgG displayed negative genetic correlations with BW72 and SL18 (r_g_ ranging from −0.15 to −0.21). However, phenotypic correlations for these trait pairs were weakly positive, mirroring the inconsistency between genetic and phenotypic correlation directions also observed in the RIR line.

Within the production traits of the WL line, highly synergistic relationships supporting high productivity were evident. AFE was positively correlated with FEW (r_g_ = 0.61; r_p_ = 0.60, *P < 0.001*), indicating that hens with later sexual maturity produced larger eggs at first egg. EP300 and EP301-560 were positively correlated (r_g_ = 0.50; r_p_ = 0.30, *P < 0.001*), demonstrating that early high production and late-phase persistency are not mutually exclusive in WL but rather act synergistically to support overall high productivity. BW-AFE exhibited negative genetic correlations with EP300 (r_g_ = −0.11) and EP301-560 (r_g_ = −0.22), suggesting a genetic resource competition between growth and development at sexual maturity and high egg production.

**Fig. 2:**
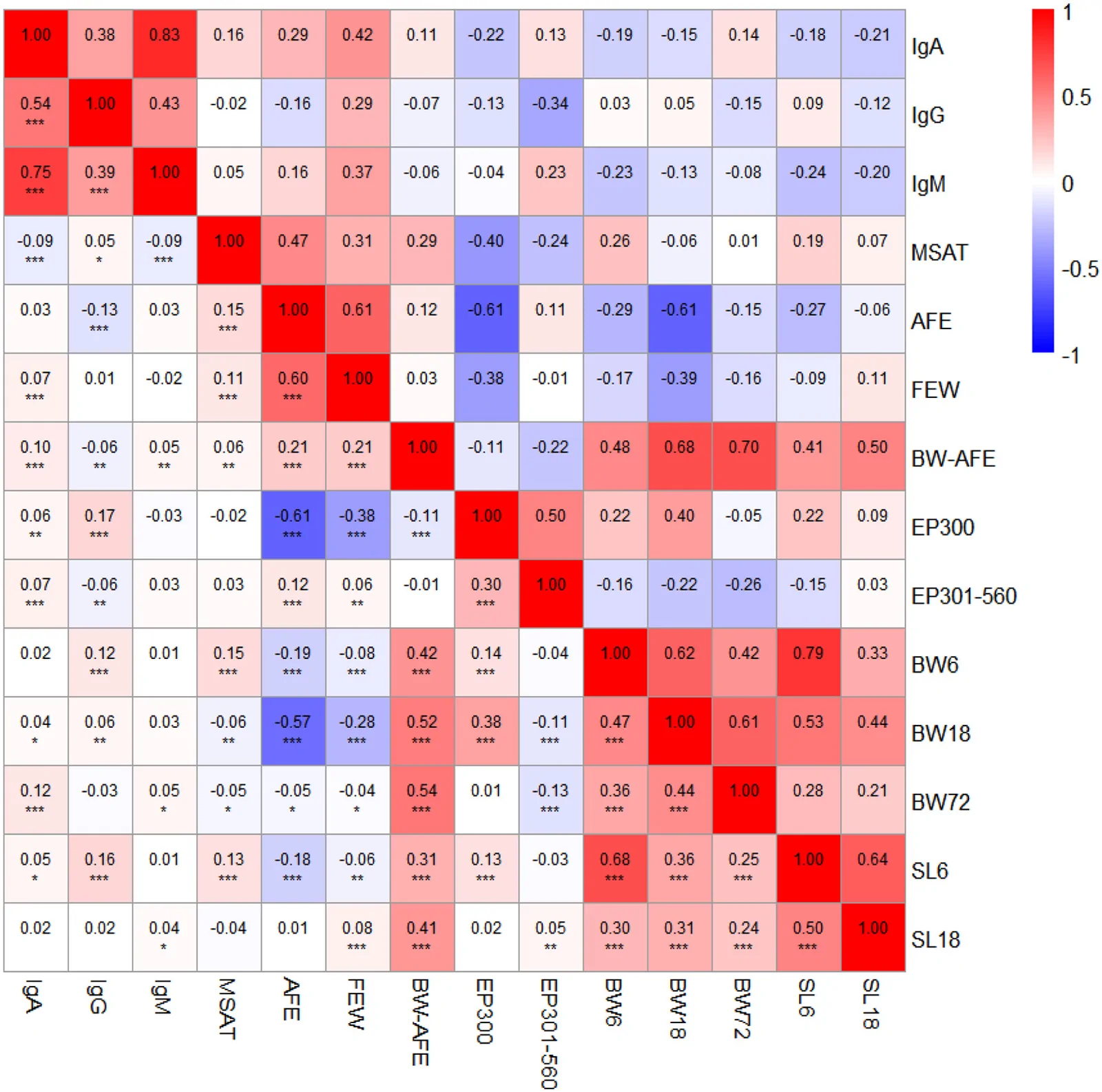
Genetic and phenotypic correlations among immune, production, and growth traits in White Leghorn (WL) hens. ^1^ Abbreviations and Definitions: IgA, IgG, IgM, concentration of immunoglobulin A, G, and M in plasma (㎍/mL); MSAT, *Mycoplasma synoviae*-specific antibody titer; AFE, age at first egg (d); FEW, egg weight at first egg (g); BW-AFE, body weight at first egg (g); EP300, total number of eggs laid from age at first egg to 300 days of age (eggs); EP301–560, total number of eggs laid from 301 to 560 days of age (eggs); BW6, BW18, BW72, body weight at 6, 18, and 72 wk of age (g); SL6, SL18, shank length at 6 and 18 wk of age (mm). ^2^ The upper triangular matrix displays genetic correlation coefficients, reflecting the genetic associations between traits. The lower triangular matrix shows phenotypic correlation coefficients, representing phenotypic associations. ^3^ Statistical significance is indicated by asterisks: * for *P* < 0.05, ** for *P* < 0.01, and *** for *P* < 0.001. ^4^ The color gradient from red to blue represents the transition of correlation coefficients from positive to negative.

Growth and developmental traits were tightly intercorrelated and exhibited synergy with early sexual maturity. Consistent with findings in the RIR line, positive correlations were observed among all body weight and shank length traits measured in the WL line. BW18 was negatively correlated with AFE (r_g_ = −0.61; r_p_ = −0.57, *P < 0.001*), mirroring the genetic association between growth rate and age at sexual maturity also observed in the RIR line. Fig. 2.

### Genetic parameter analysis of egg quality traits in the WL line

**Fig. 3:**
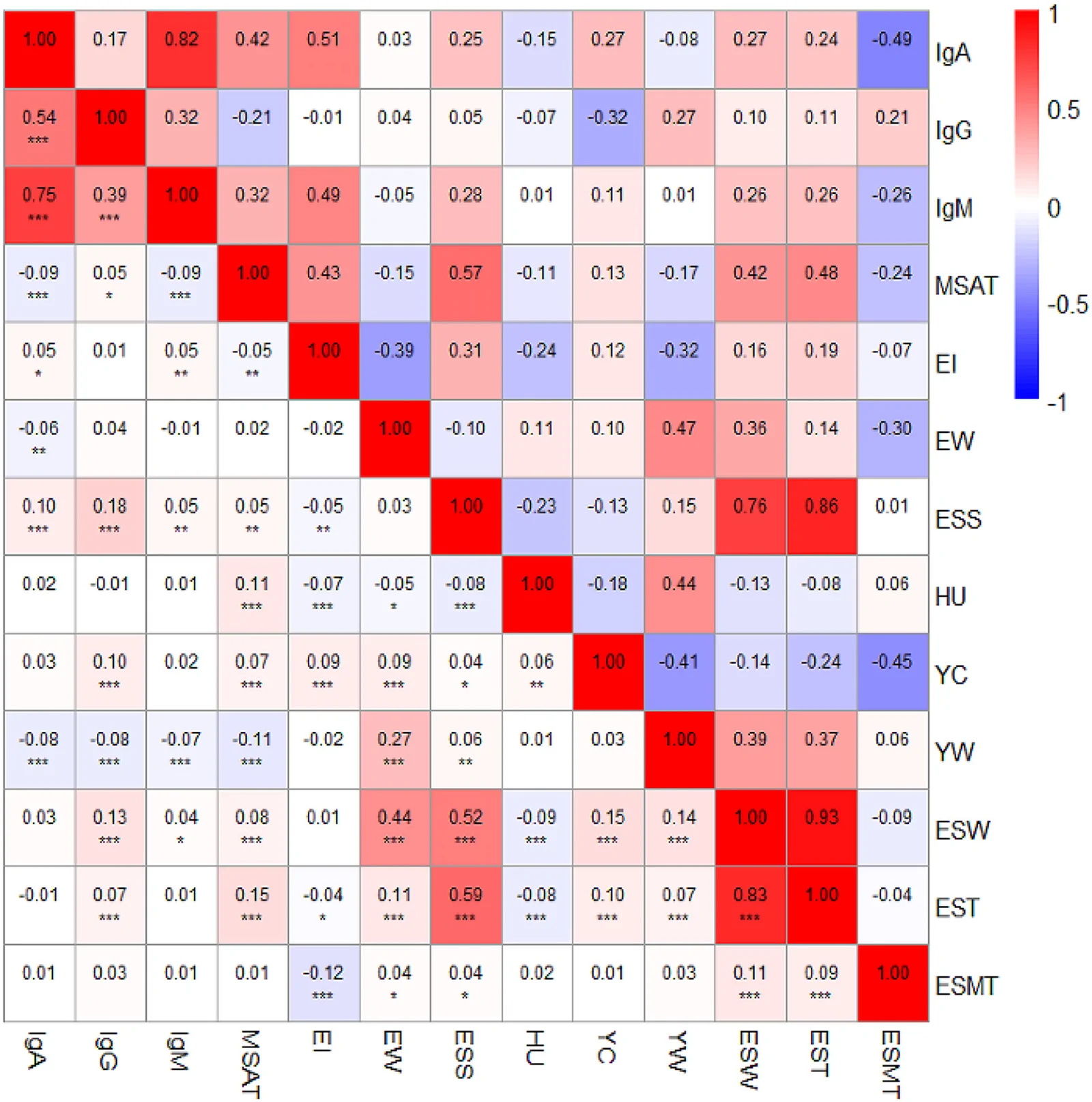
Genetic and phenotypic correlations between immune traits and egg quality traits in White Leghorn (WL) hens. ^1^ Abbreviations and Definitions: IgA, IgG, IgM, concentration of immunoglobulin A, G, and M in plasma (㎍/mL); MSAT, *Mycoplasma synoviae*-specific antibody titer; EI, egg shape index; EW, egg weight (g); ESS, eggshell strength (kg/cm²); HU, Haugh unit; YC, yolk color; YW, yolk weight (g); ESW, eggshell weight (g); EST, eggshell thickness (mm); ESMT, eggshell membrane thickness (mm). ^2^ The upper triangular matrix displays genetic correlation coefficients, reflecting the genetic associations between traits. The lower triangular matrix shows phenotypic correlation coefficients, representing phenotypic associations. ^3^ Statistical significance is indicated by asterisks: * for *P* < 0.05, ** for *P* < 0.01, and *** for *P* < 0.001. ^4^ The color gradient from red to blue represents the transition of correlation coefficients from positive to negative.

Based on the MS infection background at 100 wk of age in the WL line, genetic parameters (heritability, genetic correlations, and phenotypic correlations) were estimated for egg quality and immune traits, with results presented in Table 3 and Fig. 3. The heritability estimates for the nine egg quality traits in the WL line at 100 wk of age, under MS-specific antibody positive status, ranged from 0.06 to 0.13, indicating a low level of heritability. Among these, EW exhibited the lowest heritability (0.06 ± 0.06), while EI and YW showed the highest heritability (both at 0.13 ± 0.07).

**Table 3:** Heritability estimates of egg quality traits in White Leghorn (WL) hens.

Line Trait	WL		
	N	h²	SE
EI	1145	0.13	0.07
EW	1145	0.06	0.06
ESS	1122	0.11	0.06
HU	1054	0.12	0.07
YC	1060	0.08	0.06
YW	1069	0.13	0.07
ESW	1124	0.09	0.06
EST	1123	0.11	0.06
ESMT	1117	0.11	0.06

^1^ N represents the effective sample size for heritability calculation, h² denotes heritability, and SE stands for standard error.

^2^ EI, EW, ESS, HU, YC, YW, ESW, EST, and ESMT denote egg shape index, egg weight (g), eggshell strength (kg/cm²), Haugh unit, yolk color, yolk weight (g), eggshell weight (g), eggshell thickness (mm), and eggshell membrane thickness (mm), respectively.

MSAT exhibited positive genetic correlations with EI, ESS, ESW, and EST (r_g_ = 0.43, 0.57, 0.42, and 0.48, respectively), indicating a synergistic relationship between the genetic potential for mounting a robust antibody response to MS and the genetic capacity for producing eggs with strong, thick eggshells in WL hens. In contrast, the genetic associations between conventional humoral immunoglobulins (IgA, IgG, IgM) and egg quality traits were generally weak and heterogeneous in direction. Specifically, IgA was positively correlated with EI (r_g_ = 0.51), whereas IgG was negatively correlated with YC (r_g_ = −0.32). Furthermore, both IgA and IgM exhibited negative correlations with ESMT (r_g_ = −0.26), while IgG showed a positive correlation with ESMT (r_g_ = 0.21), reflecting the complexity underlying the genetic regulation of basal immunity.

High genetic consistency was observed among eggshell-related traits. A very strong positive genetic correlation was detected between ESS and EST (r_g_ = 0.86), and the genetic correlation between ESW and EST reached 0.93. Additionally, positive correlations were observed between EW and YW (r_g_ = 0.47) and between HU and YW (r_g_ = 0.44), indicating the presence of synergistic relationships among internal egg quality traits as well.

## Discussion

### Phenotypic characteristics of hens from the two lines

In the present study, phenotypic differences were observed between the RIR and WL lines. The WL line exhibited earlier age at first egg and superior late-phase laying persistency, whereas the RIR line displayed distinct advantages in body weight and shank length at all measured ages, as well as greater early laying peak performance. These phenotypic distinctions directly reflect the long-term divergent selection pressures applied to the RIR and WL breeds (Lewis et al., 2008; Sasaki et al., 2004). Studies on reproductive performance in RIR and WL lines by Zelleke et al. (2005) have also confirmed significant differences between these breeds. Furthermore, Sasaki et al. (2004) identified quantitative trait loci (**QTL**) affecting AFE, indicating that genetic factors associated with sexual maturity exert substantial influence on laying patterns. Bayyari et al. (1997) demonstrated that selection for different production goals results in distinct body conformation and growth patterns, and that selection for body weight and egg production is accompanied by alterations in immune and physiological responses.

In terms of immune traits, the WL line exhibited significantly higher serum IgG concentrations and MSAT. This difference may be associated with line-specific characteristics. Mo et al. (2022) reported significant differences in immune responses between early-feathering and late-feathering chickens following infection with subgroup J avian leukosis virus (**ALV-J**), indicating that even minor genetic differences controlled by a single gene can influence immune response patterns. Similarly, Kaiser et al. (2022) observed differential immune response patterns among chicken lines following Salmonella challenge. Abramoff and Brien (1968) also demonstrated that humoral immune responses in chickens are under genetic regulation. Among genetic factors, polymorphism within the major histocompatibility complex (***MHC***) has been identified as a key determinant of antibody response levels and is significantly associated with antibody titers against various antigens (Li et al., 2013). Therefore, the elevated IgG and MSAT levels observed in WL hens after MS infection may be related to the presence of MHC alleles or other immune-related genes associated with enhanced antibody responsiveness.

Beyond genetic predisposition, metabolic stress may also contribute to immune activation in WL hens. Dong et al. (2016) demonstrated that DDX21 translocates from the nucleus to the cytoplasm and stimulates innate immune responses, suggesting that metabolic stress can activate immune pathways. As a high-producing layer line, WL hens face greater metabolic demands associated with sustained egg production, and their high metabolic rate may lead to increased oxidative stress, potentially activating immune pathways.

Furthermore, all immune traits measured in the present study exhibited high phenotypic coefficients of variation. Alpert et al. (2019), through analysis of variation coefficients in immune cell subsets, revealed substantial inter- and intra-individual variation in immune traits, with variation primarily driven by non-genetic factors. Similarly, Wei et al. (2015) reported that immune traits are susceptible to environmental influences and physiological status. These findings suggest that although genetic background significantly influences immune traits, the contributions of metabolic status and environmental factors under infection conditions should not be overlooked.

### Heritability estimates for different trait categories in the two lines

Heritability is a core parameter in livestock genetic breeding, directly determining selection efficiency, and its estimates are influenced by breed, population, management environment, and statistical methods. The heritability estimates for MSAT obtained in this study provide, for the first time, a direct genetic basis for MS resistance breeding. Immune traits (IgA, IgG, IgM, MSAT) in both layer lines exhibited low heritabilities (0.09-0.19), which is highly consistent with numerous previous studies on natural antibodies or basal immunoglobulins in laying hens. Arango et al. (2024) reviewed existing studies on the heritability of natural antibodies (**NAb**) and adaptive antibodies (**AAb**, induced by vaccines or pathogens) in laying hens, reporting that antibody heritabilities generally range from 0.04 to 0.31 . Studies on chickens challenged with IBD, IBV, and NDV have reported a wide range of antibody heritability estimates (0-0.58) (Arango et al., 2024). Sun et al. (2015) found that heritabilities for various immunoglobulin isotypes (**IgT, IgM, IgA, IgG**) were consistently low (0.07-0.14) and emphasized the influence of maternal environmental effects, which is highly consistent with the findings of the present study. Asghari et al. (2025) reported that heritabilities for immune-related antibodies in Japanese quail ranged from 0.05 to 0.17, also in close agreement with our results. Berghof et al. (2013) reported moderate heritabilities (0.14-0.44) for IgM and IgG in laying hens, a range slightly higher than that observed in the present study. Furthermore, Pinard et al. (2002), based on long-term selection experiments, estimated the heritability of antibody response to sheep red blood cells (**SRBC**) in chickens at 0.18, consistent with our findings. These results indicate that, despite the use of different antigens, the heritability of certain antibody-mediated immune traits in specific populations is consistently low, further confirming the common characteristic of low heritability for humoral immune traits in laying hens. In the present study, the heritability estimate for MSAT in the RIR line (0.19) was slightly higher than that in the WL line (0.15), suggesting that the RIR line may possess greater selection potential for this trait.

AFE displayed high heritability in both lines (RIR: 0.58 ± 0.05; WL: 0.52 ± 0.05). Tongsiri et al. (2015), in a study of purebred and crossbred chickens under tropical conditions, estimated the heritability of AFE in the RIR line at 0.45, which is consistent with our findings. Tongsiri et al. (2020) reported a moderate heritability of 0.24 for AFE in Thai indigenous chickens, indicating that sexual maturity traits are under relatively strong genetic control across different chicken breeds. In the present study, FEW and egg production traits (EP300, EP301–560) exhibited low to moderate heritabilities, ranging from 0.12 to 0.39. Tongsiri et al. (2015) reported heritabilities for FEW of 0.29 in the RIR line and 0.36-0.38 in crossbred lines, indicating differences between lines. Zhang et al. (2025), in a study on Beijing-You chickens, reported heritabilities for egg production of 0.29 (at 43 wk of age) and 0.16 (at 61 wk of age), indicating that heritability estimates for egg production traits can vary with age. These studies are generally consistent with the findings of the present study.

The heritability estimate for EP300 in the WL line (0.12 ± 0.03) was substantially lower than that in the RIR line (0.32 ± 0.04). This may reflect the fixation of favorable alleles for the target trait through long-term, intensive selection, thereby reducing genetic variation. Ni et al. (2023) demonstrated that in commercially selected WL lines subjected to long-term breeding, the heritability of egg production is relatively low (approximately 0.15), while the heritability of EW is higher, ranging from 0.29 to 0.75, indicating that different traits are differentially affected by selection intensity.

### Differential correlations of MSAT with production and growth traits between the RIR and WL lines

The present study revealed a synergistic relationship between MSAT and laying persistency in the RIR line. In RIR hens, MSAT exhibited a positive correlation with late-phase egg number (EP301-560) and a negative correlation with SL6. Hinz et al. (2003) demonstrated that MS strains are capable of inducing systemic infection, leading to synovitis and airsacculitis, with all tested strains exhibiting pathogenicity, albeit with virulence ranging from high to mild. Such pathological lesions may impair chick growth and skeletal development. Furthermore, Catania et al. (2016) reported that MS infection can directly compromise the shell gland (uterus), resulting in **EAA**, reduced egg production, and significantly decreased egg weight (*P < 0.05*). Klose et al. (2022) identified that MS virulence factors, including GAPDH, ObgE, and OppF proteins, play critical roles in pathogen colonization and immunogenicity. Effective antibody responses may neutralize these virulence factors, thereby mitigating their persistent stimulation and energy expenditure in the host. Therefore, this suggests that elevated MSAT levels may effectively curtail MS infection, thereby preserving physiological resources for growth and reproduction.

In stark contrast to the RIR line, MSAT in the WL line exhibited antagonistic relationships with production traits. MSAT was negatively correlated with EP300 and EP301-560, and positively correlated with AFE, indicating that, within the WL line, individuals with genetically superior antibody responses tend to reach sexual maturity later and exhibit reduced egg production potential. This reflects a genetic resource competition between disease resistance and high productivity. Yang et al. (2008) demonstrated that lipopolysaccharide (**LPS**) injection significantly reduces feed intake and body weight gain in chickens, confirming the resource competition effect of immune activation on growth and production. As a modern high-producing layer line, WL has undergone long-term intensive selection for enhanced egg production performance. Zerjal et al. (2021) evaluated trade-offs among feed efficiency, growth, and immune responses in laying hens, revealing that lines selected for high production performance may exhibit concomitant alterations in immune responsiveness. The findings of the present study further substantiate the existence of such trade-offs. In the WL line, genetic resources appear to be predominantly allocated toward reproductive output (egg production). Bayyari et al. (1997) further confirmed that poultry selected for rapid growth rate and high egg production often exhibit reduced specific immune responses or increased disease susceptibility. This body of evidence supports the notion that intensive selection for production traits exerts negative pleiotropic effects on immune function. Genetically superior antibody responses necessitate the mobilization of finite resources (such as energy, amino acids, immune cells), which may directly compete with the resources required for sustaining peak egg production.

### Explanation for phenotypic correlations being lower than genetic correlations

In the present study, phenotypic correlations were consistently lower than their corresponding genetic correlations for all trait combinations, which is consistent with quantitative genetic theory. This phenomenon is commonly observed in livestock breeding and is primarily attributable to the diluting effect of environmental factors. The phenotypic correlation (rₚ) between two traits can be decomposed into a genetic component (r_g_) and an environmental (residual) component (rₑ), weighted by the heritabilities (h²) of the traits. In cases where environmental correlations are near zero or negative, the phenotypic correlation will be smaller than the genetic correlation. This dilution effect occurs because environmental factors introduce variation that is not under genetic control, thereby weakening the observable relationship between traits (Lynch and Walsh, 1998). Wang et al. (2006) reported that, in a study of genetic, environmental, and phenotypic correlations for spinal and hip bone phenotypes in a Chinese population, phenotypic correlations were generally lower than genetic correlations. Similarly, Hayes et al. (1984) found that, in their investigation of heritabilities and genetic and phenotypic correlations for milk casein fractions with production traits, genetic correlations were consistently higher than their phenotypic counterparts. This phenomenon, widely observed in animal breeding, highlights the importance of genetic correlations in breeding decisions.

## Conclusion

This study provides the first estimates of genetic parameters for MSAT in laying hens and reveals line-specific genetic relationships with production, growth, and egg quality traits. MSAT exhibited low heritability in both the RIR and WL lines (RIR: 0.19; WL: 0.15), indicating that genetic selection can enhance disease resistance. The genetic relationships between disease resistance and production performance were line-specific: MSAT was positively associated with improved late-phase egg production in RIR but showed genetic resource competition with egg production in WL. These findings suggest that effective breeding for disease resistance requires line-specific strategies based on precise genetic evaluation to achieve simultaneous genetic improvement of health, productivity, and egg quality traits.

## CRediT authorship contribution statement

**Xingyue Niu:** Writing – original draft, Investigation, Formal analysis, Data curation. **Qi Yang:** Investigation. **Xiaoyu Zhao:** Supervision, Resources. **Lei Shi:** Supervision, Resources. **Erying Hao:** Supervision, Resources. **Yifan Chen:** Supervision, Resources. **Lanhui Li:** Supervision, Resources. **Huage Liu:** Supervision, Resources. **Lijun Xu:** Supervision, Resources. **Rongyan Zhou:** Supervision, Resources. **Hui Chen:** Supervision, Resources. **Xiaorong Zhang:** Supervision, Methodology. **Dehe Wang:** Writing – review & editing.

## Disclosures

The authors declare that they have no known competing financial interests or personal relationships that could have appeared to influence the submission of this manuscript.

## Footnotes

Supplementary material associated with this article can be found, in the online version, at doi:10.1016/j.psj.2026.107228.
