# Supplementary material for: Strain-specific genetic relationships between Mycoplasma synoviae immunity and egg production traits in two layer lines
Source: Poult Sci. 2026 Jun 3;105(11):107228. doi: 10.1016/j.psj.2026.107228 (PMC13589140; doi:10.1016/j.psj.2026.107228)
Supplement: Supplementary file 1 [file mmc1.docx]

Table S1. Complete vaccination schedule of the experimental flock

| Day | Vaccine Type | Route (site) | Dose |
| --- | --- | --- | --- |
| 1 | Marek's Disease and Infectious Bursal Disease Vaccine, Live | Subcutaneous (neck) | 1 dose/hen |
| 1 | Newcastle Disease and Infectious Bronchitis Vaccine, Live | Ocular/nasal | 1 dose/hen |
| 7 | Newcastle Disease and Infectious Bronchitis Vaccine, Live | Ocular/nasal | 1 dose/hen |
| 7 | Avian Reovirus Vaccine, Inactivated | Subcutaneous (neck) | 0.2ml/hen |
| 10 | Newcastle Disease, Infectious Bronchitis, Avian Influenza (H9 Subtype) and Infectious Bursal Disease Vaccine, Inactivated | Subcutaneous (neck) | 0.2ml/hen |
| 14 | Infectious Laryngotracheitis and Avian Pox Vaccine, Live | Wing web vaccination | 1 dose/hen |
| 18 | Mycoplasma synoviae (MS-H) Vaccine , Live | Ocular drop | 0.03 ml/hen |
| 21 | Avian Influenza (H5+H7 Subtype) Vaccine, Inactivated | Subcutaneous (neck) | 0.3ml/hen |
| 21 | Newcastle Disease and Infectious Bronchitis Vaccine, Live | Ocular/nasal | 2 doses/hen |
| 28 | Mycoplasma Gallisepticum Vaccine, Live | Ocular | 1 dose/hen |
| 33 | Newcastle Disease, Avian Influenza and Fowl Adenovirus Vaccine, Inactivated | Subcutaneous (right breast) | 0.5ml/hen |
| 37 | Avian Coryza Trivalent Vaccine, Inactivated | Intramuscular (left wing shoulder) | 0.4ml/hen |
| 52 | Avian Influenza (H5+H7 Subtype) Vaccine, Inactivated | Subcutaneous (left breast) | 0.5ml/hen |
| 80 | Avian Coryza Trivalent Vaccine, Inactivated | Intramuscular (right wing shoulder) | 0.5ml/hen |
| 88 | Avian Influenza (H5+H7 Subtype) Vaccine, Inactivated | Subcutaneous (right breast) | 0.5ml/hen |
| 90 | Mycoplasma synoviae (YBF-MS1) Vaccine, Inactivated | Subcutaneous (neck) | 0.5 ml/hen |
| 95 | Infectious Laryngotracheitis and Avian Pox Vaccine, Live | Wing web vaccination | 1 dose/hen |
| 103 | Combined Newcastle Disease and Infectious Bronchitis Vaccine, Live | Ocular/nasal | 2 doses/hen |
| 103 | Newcastle Disease, Infectious Bronchitis, Egg Drop Syndrome and Avian Encephalomyelitis Vaccine, Inactivated | Ocular/nasal | 0.5ml/hen |
| 110 | Newcastle Disease, Avian Influenza and Fowl Adenovirus Vaccine, Inactivated | Subcutaneous (right breast) | 0.5ml/hen |
| 117 | Avian Coryza Trivalent Vaccine, Inactivated | Intramuscular (right wing shoulder) | 0.5ml/hen |
| 125 | Avian Influenza (H5+H7 Subtype) Vaccine, Inactivated | Subcutaneous (right breast) | 0.6ml/hen |
| 135 | Infectious Bursal Disease Vaccine, Inactivated | Intramuscular (wing shoulder) | 0.5ml/hen |
